# Phosphomolybdic acid supported on magnetic poly calix[4]resorcinarene-EDTA-chitosan network as a recyclable catalyst for the synthesis of 5-aroyl-NH-1,3-oxazolidine-2-ones

**DOI:** 10.1038/s41598-024-63493-y

**Published:** 2024-06-04

**Authors:** Setareh Moradi, Roya Mozafari, Mohammad Ghadermazi

**Affiliations:** Department of Chemistry, Faculty of Science, University of Kurdistan, Sanandaj, Iran

**Keywords:** α-Epoxyketones, NH-1,3-oxazolidine-2-ones, Phosphomolybdic acid, Calix[4]resorcinarene, Chitosan, EDTA, Chemistry, Nanoscience and technology

## Abstract

In this work, a novel procedure for immobilization of phosphomolybdic acid (PMA) on Magnetic polycalix[4]resorcinarene grafted to chitosan by EDTA (calix-EDTA-Cs) was reported. The heterogeneous nanocomposite (CoFe_2_O_4_@calix-EDTA-Cs@PMA) was applied an acid nanocatalyst for the synthesis of 5-aroyl-NH-1,3-oxazolidine-2-ones through the reaction of α-epoxyketones with sodium cyanate (NaOCN) in polyethylene glycol (PEG) as a green solvent under ultrasonic irradiation conditions. Some features of this work include quick reaction time, high reaction yield, easy separation of the catalyst, thermal stability, and eco-friendly.

## Introduction

Over recent years, the group of scientists has exhibited a great interest in the design and deployment of chemicals and processes that pose a minimal threat to the environment. This urge stems from a critical need to mitigate pollution and reduce environmental risks associated with chemical processes. Consequently, technological advancements and scientific research are increasingly leaning towards adopting renewable resources and reusable catalysts^1,2^.

Separation and recovery in homogeneous catalysis systems have historically been challenging processes. However, the advent of magnetic nanoparticles as catalysts heralds a significant shift in addressing these issues effectively. These nanoparticles offer many benefits including recyclability, and low reaction times owing to their super-paramagnetic behavior^3,4^. This method not only simplifies isolation but also augments the efficacy of the catalyst recovery process, which in turn enhances the purity of the end products^5–7^. Therefore, magnetic separation occurs as an intriguing alternative to filtration or centrifugation, because it reduces loss of catalyst and makes the recovery and reusability of catalyst easier by magnetic force after the reaction^8–12^.

Polyoxometalates (POMs) are a class of solid acid catalysts that have garnered substantial attention due to their potential to serve as economically and environmentally friendly catalysts. Characterized by their unique structural and electronic properties, POMs have been instrumental in various fields including medicine, separation science, and catalysis. Furthermore, these compounds are known to be non-porous solid acids, possessing a low surface area which restricts their efficacy in catalytic processes. Moreover, their high solubility in water and various solvents poses a challenge in separating them from reaction products, often leading to significant losses during recycling processes^13,14^.

To counter these drawbacks, researchers have embarked on strategies to enhance the stability of POMs. One effective approach has been the immobilization of POMs onto materials with high surface areas, which not only increases their efficacy but also facilitates easier separation from reaction mixtures, thus minimizing loss during recycling^15^. Several materials have been identified as suitable candidates for this process. These include active carbon^16^, chitosan^17^, GO^18^, MCM-41^19^, polymers^20^, and ZrO_2_^21^.

In the present study, chitosan was selected as a ligand owing to its green and readily accessible nature. As a renewable and natural polymer, it possesses several commendable features including biocompatibility, ease of modification, excellent chemical resistance, non-toxicity, and a favorable permselectivity towards water^22–24^. This biopolymer is derived from the deacetylation of chitin, a process that enhances its utility in various applications. The presence many hydroxyl and amino groups on the surface of its polymer chains positions it as a pivotal player in chemical modifications occurring on the chitosan surface, thereby broadening its potential roles and applications in environmental science and technology^25–28^.

Indeed, to optimize its utility further, it is sometimes required to enhance the catalytic efficiency of chitosan by incorporating active metallic species or by grafting appropriate functional groups onto it. In addition, utilizing a Polymer network in 3D formed by calix[4]resorcinarene stands as a promising strategy in the advancement of porous organic polymers, particularly in the realm of surface modification^29–31^. The concurrent application of chitosan and poly calix[4]resorcinarene in the formulation of heterogeneous catalysts potentially leads to the creation of materials with superior mechanical and thermal properties, all while being cost-effective^32,33^.

*N*-Heterocyclic compounds stand as some of the most adaptable building blocks in medicinal chemistry, serving as the backbone in the formulation of numerous potent bioorganic compounds. Particularly, oxazolidin derivatives, especially those containing the NH-oxazolidin-2-one moiety, have been pivotal in advancing the development of various biological combinations. These derivatives exhibit a range of valuable activities comprising anti-cancer, anti-inflammatory, antipyretic, anticonvulsant, antifungal, antimicrobial, anthelmintic, antianxiety, and anti-depressive effects, as well as acting as HIV-1 inhibitors. Their versatility makes them critical assets in pharmaceutical drug design^34,35^.

Building on our recent efforts to employ heterogeneous nanocatalysts in the synthesis of NH-oxazolidin-2-ones, we are eager to present our findings on utilizing CoFe_2_O_4_@calix-EDTA-Cs@PMA as a green acid nanocatalyst. This novel approach facilitates the eco-friendly synthesis of a fresh series of NH-1,3-oxazolidine-2-ones derivatives through a one-pot reaction involving α-epoxyketones and NaOCN, undertaken in the presence of polyethylene glycol at a moderate temperature of 50 °C under ultrasonic conditions.

## Experimental

### Materials and instrumentation

All the chemicals and solvents utilized for the synthesis of CoFe_2_O_4_@calix-EDTA-Cs@PMA, including CoCl_2_⋅6H_2_O, FeCl_3_·6H_2_O, Chitosan, resorcinol, phosphomolybdic acid were bought from the companies Merck and Sigma Aldrich. α-epoxyketones was prepared according to the literature.

The instruments used for the characterization of CoFe_2_O_4_@calix-EDTA-Cs@PMA included XRD, FT-IR, TGA, EDX, AFM, VSM, BET, ICP-OES and SEM techniques.

The structure of the nanocatalyst was displayed using a scanning electron microscope by FESEM-TESCAN MIRA3. XRD data were recorded using Cu kα radiation (λ = 1.54 Å) within the region of 2ϴ = 20°–80°. Fourier Transform Infrared Spectroscopy were taken on a VRTEX 70 model BRUKER spectrophotometer in potassium bromide discs and reported in cm^−1^. Thermogravimetric analysis information for the nanocatalyst was recorded on a Shimadzu DTG-60, with a maximum heating rate of 10 °C/min. Inductively Coupled Plasma Optical Emission Spectrometry (ICP-OES) and Energy-dispersive X‐ray spectroscopy analysis was by MIRA3TESCANXMU instrument for elemental analysis. Vibrating sample magnetometer measurement was recorded by Meghnatis Daghigh Kavir Company. The pore size distribution and surface area were investigated using Barrett–Joyner–Halenda (BJH) analysis and Brunauer–Emmett–Teller (BET) measurements, respectively. Atomic force microscopy observations were conducted using tapping-mode on the Nano Wizard II atomic force microscope. The checking of product purity and monitoring of reactions were done using TLC on silica gel polygram SIL G/UV254 plates. ^1^H and ^13^C NMR spectra were measured on a Bruker 250 MHz spectrometer in CDCl_3_ with chemical shift (δ) given in ppm. Mass spectrometer operating at an ionization potential of 70 eV.

### Synthesis of calix[4]resorcinarene

Calix[4]resorcinarene is prepared according to the mentioned method^36^. In brief, a mixture of 70 mL HCl (37%) and 70 mL distilled water was combined with 70 mL of ethanol containing 14 g of resorcinol in a three-necked bottom (250 mL) under argon gas. After that, under mechanical stirring, 0.14 mol of acetaldehyde was slowly dripped into the solution within 20 min, after being mixed for 15 min, the solution was then heated to 50 °C and kept at that temperature for 1 h. The reaction mixture was cooled until it reached room temperature and stirred under N_2_ atmosphere for 48 h. Finally, the sediments obtained were rinsed multiple times with double-distilled water and placed in an oven at 70 °C for 12 h.

### Synthesis of the porous organic polymer based on calix[4]resorcinarene

To synthesize the poly calix[4]resorcinarene, 40 mL of NaOH solution (10%) was added to a three-necked bottom containing 7.2 g of calix[4]resorcinarene prepared in “Synthesis of calix[4]resorcinarene” section, under argon gas. 40 mmol of formaldehyde was added dropwise to the resulting red solution during a 30 min time frame. The combination was stirred non-stop for 20 h at 90 °C. Once the reaction had been finished, the sediment obtained was rinsed twice with distilled water. Next, the resulting gel was stirred in 55 mL of Hydrochloric acid solution (0.1 M) for 50 min. Finally, the filtered polymer was rinsed multiple times with distilled water and dried at 90 °C for 10 h.

### Synthesis of the magnetic CoFe_2_O_4_ nanoparticles

Cofe_2_o_4_ is prepared according to the mentioned method^37^. In a three-necked flask, 6.46 g of Fe (NO_3_)_3_⋅9H_2_O and 2.32 g of Co(NO_3_)_2_⋅6H_2_O were dissolved in 40 mL of distilled water, with the blend vigorously stirred at ambient temperature. Sodium hydroxide (3 M) was added dropwise until the pH reached 11–12. The stirring continued at 80 °C for 1 h. The synthesized CoFe_2_O_4_ nanoparticles were separated from the reaction mixture with a magnet and washed repeatedly with hot distilled water and ethanol to eliminate impurities.

### Synthesis of CoFe_2_O_4_@calix nanocatalyst

To prepare the CoFe_2_O_4_@calix nanocatalyst, poly calix[4]resorcinarene was first synthesized as outlined in the given reference^36^. Subsequently, 1.0 g of the poly calix[4]resorcinarene was solubilized in 20 mL of distilled water. Then, 1.0 g of CoFe_2_O_4_ nanoparticles prepared in “Synthesis of the magnetic CoFe_2_O_4_ nanoparticles” section were added to this solution and were consistently stirred at room temperature for 24 h. The resulting nanocomposite was then isolated using an external magnet and washed over and over with a mixture of distilled water and ethanol. The final step involved drying the material in the oven set at 60 °C for a duration of 10 h.

### Synthesis of CoFe_2_O_4_@calix-EDTA-Cs@PMA

In a round bottomed (250 mL), 1.0 g of chitosan was solubilized in 12 mL of acetic acid solution (0.05 M). Subsequently, 1.0 g of magnetic poly calix[4]resorcinarene was integrated into the blend, which was then mixed for 30 min. Following this, 8.0 g of the EDTA dianhydride cross-linker, prepared as per the Tülü and Geckeler method^22^, was incorporated under mechanical agitation. The reaction mixture was stirred at 70 °C for a duration of 10 h, during which the cross-linking of the polymer coated with chitosan took place. The subsequent stage of CoFe_2_O_4_@calix-EDTA-Cs@PMA synthesis, 4.5 g of phosphomolybdic acid (H_3_PMo_12_O_40_) was dissolved in 100 mL of distilled water. Subsequently, this solution was added to the previously mentioned blend and agitated at 60 °C for 2 h. The CoFe_2_O_4_@calix-EDTA-Cs@PMA catalyst was then isolated using an external magnet and washed multiple times with distilled water to eliminate any excess phosphomolybdic acid, and dried at 50 °C for 12 h.

### Synthesis of NH-oxazolidin-2-ones in the presence of CoFe_2_O_4_@calix-EDTA-Cs@PMA

1 mmol of α-epoxy ketone and 4 mmol of PEG-400 were introduced into a three-necked flask. To this mixture, 25 mg of CoFe_2_O_4_@calix-EDTA-Cs@PMA catalyst was added. The flask was then placed in an ultrasonic bath at room temperature for approximately 3 min. Subsequently, 1 mmol of NaOCN was introduced, and the mixture was subjected to ultrasonic conditions at 50 °C for the time periods specified in Table 1. The progress of the reaction was monitored through thin layer chromatography (TLC). After finishing, the catalyst was effortlessly separated from the reaction mixture using an external magnet.

**Table 1:** The optimization of reaction parameters for the synthesis of NH-1,3 oxazolidin-2-one.


Entry	Amount of catalyst (mg)	Reaction Condition	Solvent	Time (min)	Yield (%)^a^
1	25	80 °C	CH_3_CN	90	43
2	25	80 °C	DMSO	90	82
3	25	80 °C	*n*-Hexane	90	26
4	25	80 °C	EtOH	90	83
5	25	80 °C	H_2_O	90	45
6	25	80 °C	PEG-400	45	90
7	25	Sonication/50 °C	PEG-400	30	95
8	25	Sonication/60 °C	PEG-400	30	93
9	25	Sonication/40 °C	PEG-400	40	89
10	25	Sonication/r.t	PEG-400	60	61
11	3	Sonication/50 °C	PEG-400	30	93
12	2	Sonication/50 °C	PEG-400	45	88
13	15	Sonication/50 °C	PEG-400	45	79
14	–	Sonication/50 °C	PEG-400	60	–
15	PMA (25)	Sonication/50 °C	PEG-400	60	83
16	CoFe_2_O_4_ (25)	Sonication/50 °C	PEG-400	60	45

Reaction conditions: α-epoxyketone (1 mmol), sodium cyanate (1 mmol), CoFe_2_O_4_@calix-EDTA-Cs@PMA (25 mg,), solvent (4 mL).

^a^Isolated yield.

Te structure of each purifed compound was confrmed with a comparison of their FT-IR, ^1^HNMR, ^13^C NMR, and Mass spectra with authentic samples.

#### *cis*-5-Benzoyl-4-phenyloxazolidin-2-one (*cis*-2a)

IR (liquid film): *ν* (cm^−1^) = 3465 (NH), 1726 (CO-Carbamate), 1687 (CO-Ketone).

^1^H NMR (250 M Hz, CDCl_3_): *δ* = 8.51 (d, ^3^*J*_HH_ = 7.2 Hz, 2H–Ar), 8.04 (dd, ^3^*J*_HH_ = 7.8, 7.0 Hz, 2H-Ar), 7.78–7.70 (m, 4H–Ar), 7.55 (dd, ^3^*J*_HH_ = 7.5, ^4^*J*_HH_ = 1.6 Hz, 2H–Ar), 6.76 (d, ^3^*J*_HH_ = 2.8 Hz, C^5^–H), 6.09 (d, ^3^*J*_HH_ = 2.8 Hz, C^4^–H), 4.17 (brs, NH) ppm.

^13^C NMR (69.2 MHz CDCl_3_): *δ* = 197.8, 160.9, 135.1, 134.2, 133.5, 129.7, 129.4, 129.3, 128.6, 127.9, 78.2, 75.4 ppm.

EI-MS (70 eV): *m/z* (%) = 267 (M ^o+^, 5), 239 (9), 224 (10), 223 (8), 208 (19), 105 (100), 77 (62).

#### *trans*-5-Benzoyl-4-phenyloxazolidin-2-one (*trans*-2a)

IR (liquid film): *ν* (cm^−1^) = 3456 (NH), 1721 (CO-Carbamate), 1686 (CO-Ketone).

^1^H NMR (250 M Hz, CDCl_3_): *δ* = 8.04 (dd, ^3^*J*_HH_ = 6.7, Hz, ^4^*J*_HH_ = 1.6 Hz, 2H–Ar), 7.57 (dd, ^3^*J*_HH_ = 8.0, 7.5 Hz, 2H–Ar), 7.27–7.24 (m, 4H–Ar), 7.08 (dd, ^3^*J*_HH_ = 7.5 Hz, ^4^*J*_HH_ = 1.6 Hz, 2H–Ar), 6.29 (d, ^3^*J*_HH_ = 2.0 Hz, C^5^–H), 5.62 (d, ^3^*J*_HH_ = 2.0 Hz, C^4^–H), 3.68 (bs, NH) ppm.

^13^C NMR (69.2 MHz CDCl_3_): *δ* = 197.3, 160.4, 134.7, 133.8, 133.0, 129.3, 128.9, 128.9, 128.1, 127.4, 76.6, 75.0 ppm.

EI-MS (70 eV): *m/z* (%) = 267 (M^o+^, 3), 239 (8), 224 (7), 223 (5), 208 (15), 105 (100), 77 (72).

#### cis-5-Benzoyl-4-(4-methylphenyl) oxozolidin-2-one (cis-2b)

IR (liquid flim): *ν* (cm^−1^) = 3445 (NH), 1727 (CO-Carbamate), 1686 (CO-Ketone).

^1^H NMR (250 MHz, CDCl_3_): *δ* = 8.03 (dd, ^3^*J*_HH_ = 8.0 Hz, ^4^*J*_HH_ = 1.5 Hz, 2H–Ar), 7.70 (dd, ^3^*J*_HH_ = 7.2 Hz, ^4^*J*_HH_ = 1.5 Hz, 1H–Ar), 7.58 (dd, ^3^*J*_HH_ = 8.0 Hz, ^3^*J*_HH_ = 7.2 Hz, 2H–Ar), 7.05 (d, ^3^*J*_HH_ = 8.0 Hz, 2H–Ar), 6.96 (d, ^3^*J*_HH_ = 8.0 Hz, 2H–Ar), 6.25 (d, ^3^*J*_HH_ = 3.0 Hz, C^5^–H), 5.61 (m, C^4^–H), 3.67 (d, ^3^*J*_NH_ = 6.5 Hz, NH), 2.31 (s, CH_3_) ppm.

^13^C NMR (69.2 MHz CDCl_3_): *δ* = 197.4, 160.4, 138.8, 134.6, 133.7, 129.9, 129.2, 128.8, 127.4 (2C), 75.0 (2C), 21.2 ppm.

EI-MS (70 eV): *m/z* (%) = 281 (M^o+^, 4), 253 (4), 237 (13), 222 (15), 105 (100), 91 (67), 77 (31).

#### trans-5-Benzoyl-4-(4-methylphenyl)oxozolidin-2-one (trans-2b)

IR (liquid flim): *ν* (cm^−1^) = 3460 (NH), 1719 (CO-Carbamate), 1686 (CO-Ketone).

^1^H NMR (250 MHz, CDCl_3_): *δ* = 7.90 (dd, ^3^*J*_HH_ = 8.2 Hz, ^4^*J*_HH_ = 1.5 Hz, 2H–Ar), 7.53 (dd, ^3^*J*_HH_ = 7.7 Hz, ^4^*J*_HH_ = 1.5 Hz, 1H–Ar), 7.33 (d, ^3^*J*_HH_ = 8.2 Hz, 2H–Ar), 7.28 (d, ^3^*J*_HH_ = 7.7 Hz, 2H–Ar), 7.16 (d, ^3^*J*_HH_ = 8.0 Hz, 2H–Ar), 6.17 (d, ^3^*J*_HH_ = 2.5 Hz, C^5^–H), 5.33 (m, C^4^–H), 3.89 (bs, NH), 2.33 (s, CH_3_) ppm.

^13^C NMR (69.2 MHz CDCl_3_): *δ* = 198.1, 159.4, 138.6, 134.3, 133.4, 129.2, 129.0, 128.6, 127.0, 126.4, 75.4, 75.3, 21.2 ppm.

EI-MS (70 eV): *m/z* (%) = 281 (M^o+^, 5), 253 (6), 237 (10), 222 (9), 105 (100), 91 (77), 77 (23).

#### 5-(4-Methylbenzoyl)-4-(4-methylphenyl)oxozolidin-2-one (Mixture of *cis* and *trans*-2c)

IR (KBr): *ν* (cm^−1^) = 3455 (NH), 1718 (CO-Carbamate), 1676 (CO-Ketone).

^1^H NMR (250 MHz, CDCl_3_): *δ* = 7.96 (d, ^3^*J*_HH_ = 8.0 Hz, 2H–Ar), 7.80 (d, ^3^*J*_HH_ = 8.0 Hz, 2H–Ar), 7.37 (d, ^3^*J*_HH_ = 7.7 Hz, 2H–Ar), 7.31 (m, 4H–Ar), 7.16 (d, ^3^*J*_HH_ = 8.0 Hz, 2H–Ar), 7.02 (2d, ^3^*J*_HH_ = 7.7 Hz, ^3^*J*_HH_ = 8.0 Hz, 4H–Ar), 6.25 (s, *cis*-C^5^–H), 6.15 (s, *trans*-C^5^–H), 5.58 (s, *cis*-C^4^–H), 5.30 (s, *trans*-C^4^–H), 395–3.69 (bs, 2NH), 2.50, 2.30 (2s, 2*cis*-CH_3_), 2.44, 2.34 (2s, 2*trans*-CH_3_) ppm.

^13^C NMR (69.2 MHz, CDCl_3_): *δ* = 196.8, 195.6, 160.4, 159.4, 145.9, 138.7, 138.0, 131.2, 130.0, 129.7, 129.4, 129.2, 129.0, 128.8, 128.7, 128.6, 127.6, 127.5, 127.4, 127.0, 76.6, 75.5, 75.2, 74.9, 21.9, 21.8, 21.3, 21.2 ppm.

EI-MS (70 eV): *m/z* (%) = 295 (M ^o+^, 3), 267 (1), 150 (21), 119 (100), 105 (14), 91 (77), 77 (23).

#### cis-5-(4-Methoxybenzoyl)-4-phenyloxozolidin-2-one (cis-2d)

IR (liquid film): *ν* (cm^−1^) = 3455 (NH), 1720 (CO-Carbamate), 1673 (CO-Ketone).

^1^H NMR (250 MHz, CDCl_3_): *δ* = 8.06 (d, ^3^*J*_HH_ = 7.0 Hz, 2H–Ar), 7.30 (d, ^3^*J*_HH_ = 8.2 Hz, 2H–Ar), 7.26 (d, ^3^*J*_HH_ = 7.5 Hz, 2H–Ar), 7.11 (dd, ^3^*J*_HH_ = 7.2 Hz, ^4^*J*_HH_ = 1.5 Hz, 1H–Ar), 7.08 (d, ^3^*J*_HH_ = 7.0 Hz, 2H–Ar), 6.28 (d, ^3^*J*_HH_ = 2.5 Hz, C^5^–H), 5.56 (bs, C^4^–H), 3.93 (s, OCH_3_), 3.75 (bs, NH) ppm.

^13^C NMR (69.2 MHz CDCl_3_): *δ* = 195.3, 164.7, 160.5, 133.8, 129.9, 129.3, 128.9, 128.1, 127.5, 114.5, 76.8, 74.6, 55.7 ppm.

EI-MS (70 eV): *m/z* (%) = 297 (M^o+^, 1), 254 (27), 253 (7), 135 (100), 107 (17), 105 (18), 77 (44).

#### cis-5-(4-Methoxybenzoyl)-4-(4-methoxyphenyl)oxozolidin-2-one (cis-2e)

IR (liquid film): *ν* (cm^−1^) = 3450 (NH), 1719 (CO-Carbamate), 1676 (CO-Ketone).

^1^H NMR (250 MHz, CDCl_3_): *δ* = 8.07 (d, ^3^*J*_HH_ = 8.5 Hz, 2H–Ar), 7.05–7.00 (m, 4H–Ar), 6.77 (d, ^3^*J*_HH_ = 8.5 Hz, 2H–Ar), 6.23 (d, ^3^*J*_HH_ = 3.0 Hz, C^5^–H), 5.54 (dd, ^3^*J*_NH_ = 6.5 Hz, ^3^*J*_HH_ = 3.0 Hz, C^4^–H), 3.94 and 3.77 (2s, 2OCH_3_), 3.88 (d, ^3^*J*_NH_ = 6.5 Hz, NH) ppm.

^13^C NMR (69.2 MHz CDCl_3_): *δ* = 196.1, 164.7, 160.5, 131.3, 128.9, 126.5, 125.1, 118.7, 114.5, 113.5, 77.2, 74.6, 55.6, 55.2 ppm.

EI-MS (70 eV): *m/z* (%) = 284 (M^o+^–HNCO, 5), 283 (4), 177 (13), 135 (100), 107 (47).

#### trans-5-Benzoyl-4-(2-methoxyphenyl)oxozolidin-2-one (trans-2f)

^1^H NMR (250 MHz, CDCl_3_): *δ* = 8.1 (dd, ^3^*J*_HH_ = 7.0 Hz, ^4^*J*_HH_ = 1.2 Hz, 2H–Ar), 7.66 (dd, ^3^*J*_HH_ = 7.5 Hz, ^3^*J*_HH_ = 7.2 Hz, 1H–Ar), 7.53 (dd, ^3^*J*_HH_ = 7.5 Hz, ^3^*J*_HH_ = 7.2 Hz, 2H–Ar), 7.40 (d, ^3^*J*_HH_ = 7.5 Hz, 1H–Ar), 7.31 (m, 1H–Ar), 7.02 (2d, ^3^*J*_HH_ = 7.5 Hz, ^3^*J*_HH_ = 7.5 Hz, 1H–Ar), 6.90 (d, ^3^*J*_HH_ = 8.5 Hz, 1H–Ar), 6.64 (s, C^5^–H), 5.42 (s, C^4^–H), 3.99 (s, OCH_3_), 3.89 (bs, NH) ppm.

C NMR (69.2 MHz, CDCl_3_): *δ* = 198.3, 159.2, 155.2, 134.3, 133.3, 129.3, 128.9, 128.7, 127.2, 124.7, 120.9, 110.2, 73.9, 70.9, 55.5 ppm.

EI-MS (70 eV): *m/z* (%) = 297 (M^o+^, 2), 254 (23), 107 (25), 105 (100).

## Results and discussion

### Characterization of CoFe_2_O_4_@calix-EDTA-Cs@PMA nanocatalyst

**Scheme 1:**
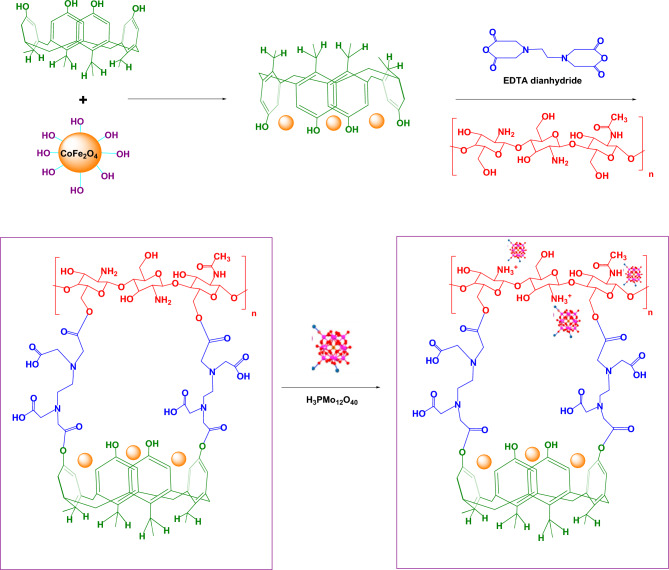
Synthesis CoFe_2_O_4_@calix-EDTA-Cs@PMA catalyst.

The method used to make CoFe_2_O_4_@calix-EDTA-Cs@PMA is outlined in Scheme 1.

Initially, a 3D-network porous polymer was crafted using a series of steps. This began with the reaction of resorcinol with acetaldehyde, subsequently leading to the polycondensation of calix[4]resorcinarene with formaldehyde. Concurrently, CoFe_2_O_4_ nanoparticles were synthesized using a co-precipitation technique and later melded with the 3D-network polymers. This union was fortified by the robust hydrogen bond between CoFe_2_O_4_, oxygen and the hydroxyl group in the polymers. Intricacies arise from chitosan’s acetamide or primary amino groups in the (*N*-acetyl-) d-glucosamine unit, enabling unique conformational properties through both intra and intermolecular hydrogen bonding. Moreover, the residual amine and hydroxyl groups in both chitosan and the 3D-network polymers serve as versatile functional groups. These groups facilitate diverse chemical modifications, allowing for the emergence of derivatives with distinct characteristics. The magnetic 3D-network polymer surface was further tailored by grafting it with EDTA and the free amines from chitosan. These nucleophilic functional groups target the carbonyl groups in EDTA, resulting in the formation of a polymer layer over the magnetic polymer surface. Concurrently, H_3_PMo_12_O_40_ molecules are anchored to this polymer layer by reacting with it. This novel polymer layer acts as a protective shield, isolating the anchored H_3_PMo_12_O_40_ from the base CoFe_2_O_4_. This layer is pivotal in thwarting unwanted agglomeration, leakage, and dissolution of CoFe_2_O_4_ nanoparticles, especially in acidic environments, ensuring its integrity.

#### FT-IR spectroscopy

**Figure 1:**
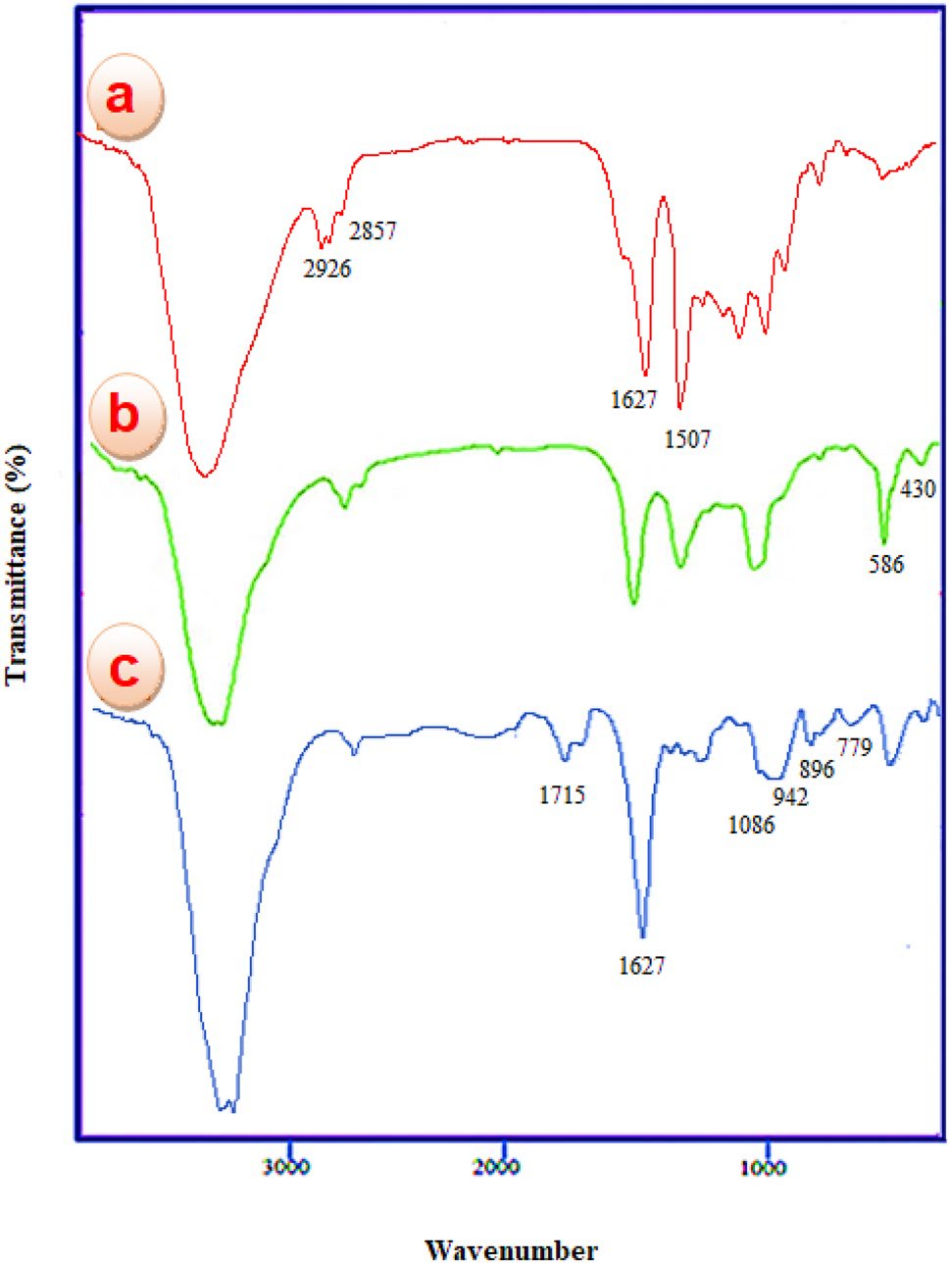
FT-IR spectra of poly calix[4]resorcinarene (**a**), CoFe_2_O_4_@calix (**b**), CoFe_2_O_4_@calix-EDTA-Cs@PMA (**c**).

As depicted in Fig. 1, the FT-IR technique serves as a tool to describe and verify the successful synthesis of the catalyst. Comparing the FT-IR spectra of poly calix[4]resorcinarene (a) CoFe_2_O_4_@calix (b), and CoFe_2_O_4_@calix-EDTA-Cs@PMA (c), the broad band observed within the bounds of 3100–3500 cm^−1^ is indicative of the hydroxyl functional group (O–H) present in the 3D-Network polymers. Stretching vibration of C–H group was characterized by a Peaks in the range of 2800–2900 cm^−1^. Moreover, the phenyl rings in all structures are shown by the Peaks at 1507 and 1617 cm^−1^ (Fig. 1a)^38^. Stretching vibrations of Fe–O bonds in areas 430 cm^−1^ and 586 cm^−1^ appear in the tetrahedral and octahedral sites of CoFe_2_O_4_ respectively (Fig. 1b)^39^. Furthermore, in the FTIR spectra of CoFe_2_O_4_@calix-EDTA-Cs@PMA, distinct absorption peaks emerge at 1627 cm^−1^, 1685 cm^−1^, and 1715 cm^−1^, which are attributed to the amide, acid, and ester functional groups, respectively. These findings signify the successful grafting of 3D-Network polymers onto chitosan via EDTA.

Additionally, the unmistakable peaks associated with this catalyst are located at 1086 cm^−1^, 942 cm^−1^, 896 cm^−1^, and 779 cm^−1^. These are representative bands corresponding to νas (Mo–O), νas (Mo = O), νas (Mo–Ob–Mo), and νas (Mo–Oc–Mo) of the Keggin-like PMA structure. This evidence points towards the successful incorporation of PMA species within the magnetic Network polymers facilitated by chitosan multilayers, which are rich in amino groups (Fig. 1c).

#### X-ray diffraction

**Figure 2:**
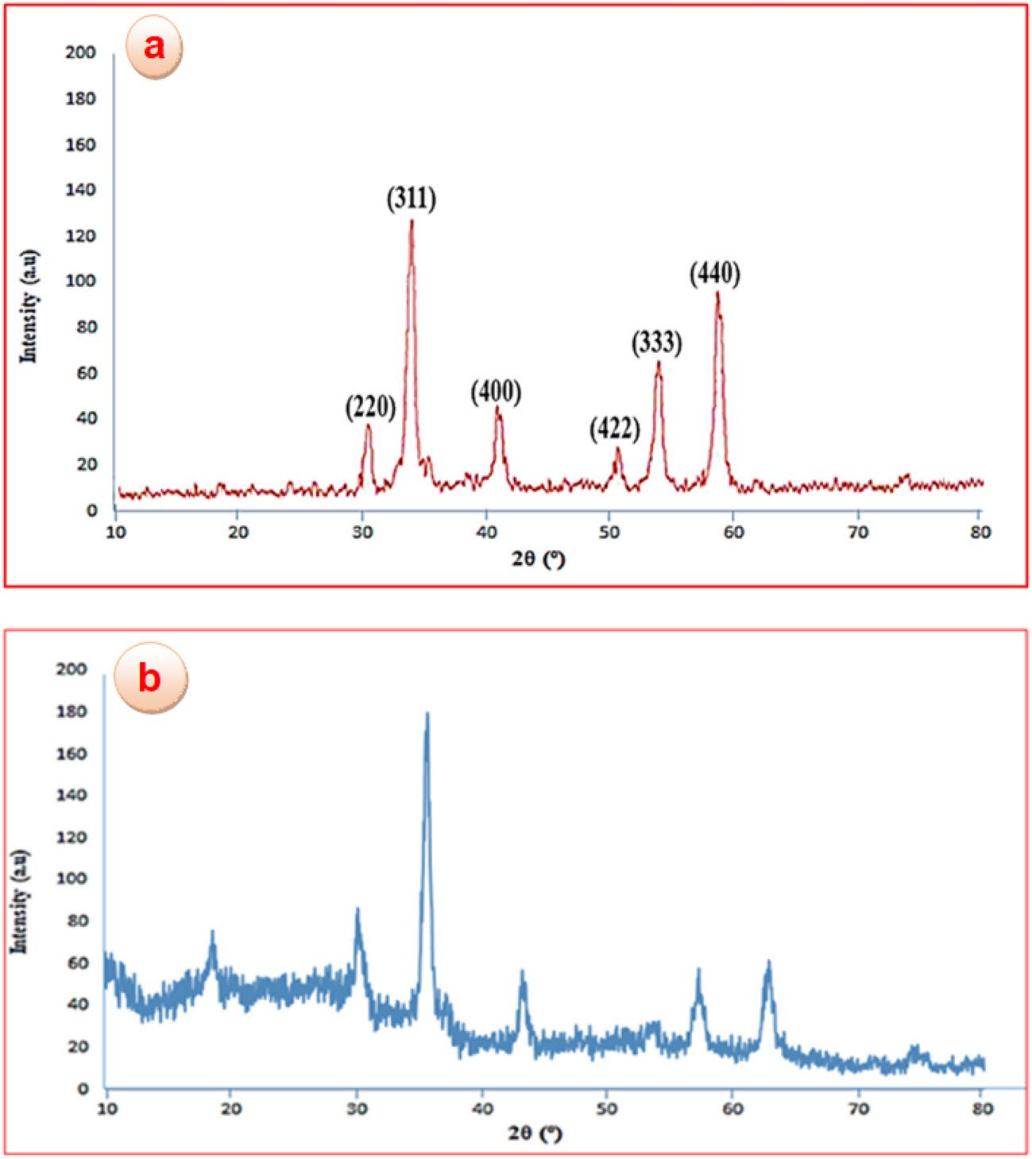
X-ray diffraction (XRD) pattern of CoFe_2_O_4_ (**a**) and CoFe_2_O_4_@calix-EDTA-Cs@PMA (**b**).

X-ray diffraction patterns (XRD) of the CoFe_2_O_4_ (a) and the CoFe_2_O_4_@calix-EDTA-Cs@PMA (b) are presented in Fig. 2. The diffraction peaks related to Bragg’s reflections from (220), (311), (400), (422), (333), and (440) planes correspond to the standard spinel structure of CoFe_2_O_4_ (JCPDS card No. 22-1086)^36^. Based on the Debye–Scherrer equation, the mean size of this CoFe_2_O_4_ particle is calculated to be nearly 11 nm (Fig. 2a). Also, the successful synthesis of CoFe_2_O_4_@calix-EDTA-Cs@PMA was further confirmed by XRD patterns as well. As depicted, according to the standard XRD data (JCPDS card No. 01-0032) peaks at 2θ = 28.12°, 38.59°, 46.56°, 53.02°, 57.75°, 61.80°, and 75.60°, ascribed to the PMA^40^. Moreover, the diffraction peak in the chitosan was observed at 20.3° of as-synthesized CoFe_2_O_4_@calix-EDTA-Cs@PMA nanocatalyst^41^.

This evidence underscores the successful grafting of the magnetic 3D-network polymers onto the chitosan backbone, facilitated by EDTA acting as a bidentate cross-linker. The presence of these peaks substantiates the uniform coverage of chitosan multilayers, the optimal dispersion of PMA, and the fine nanocrystalline character of the organic magnetic components (Fig. 2b).

#### VSM analysis studies

**Figure 3:**
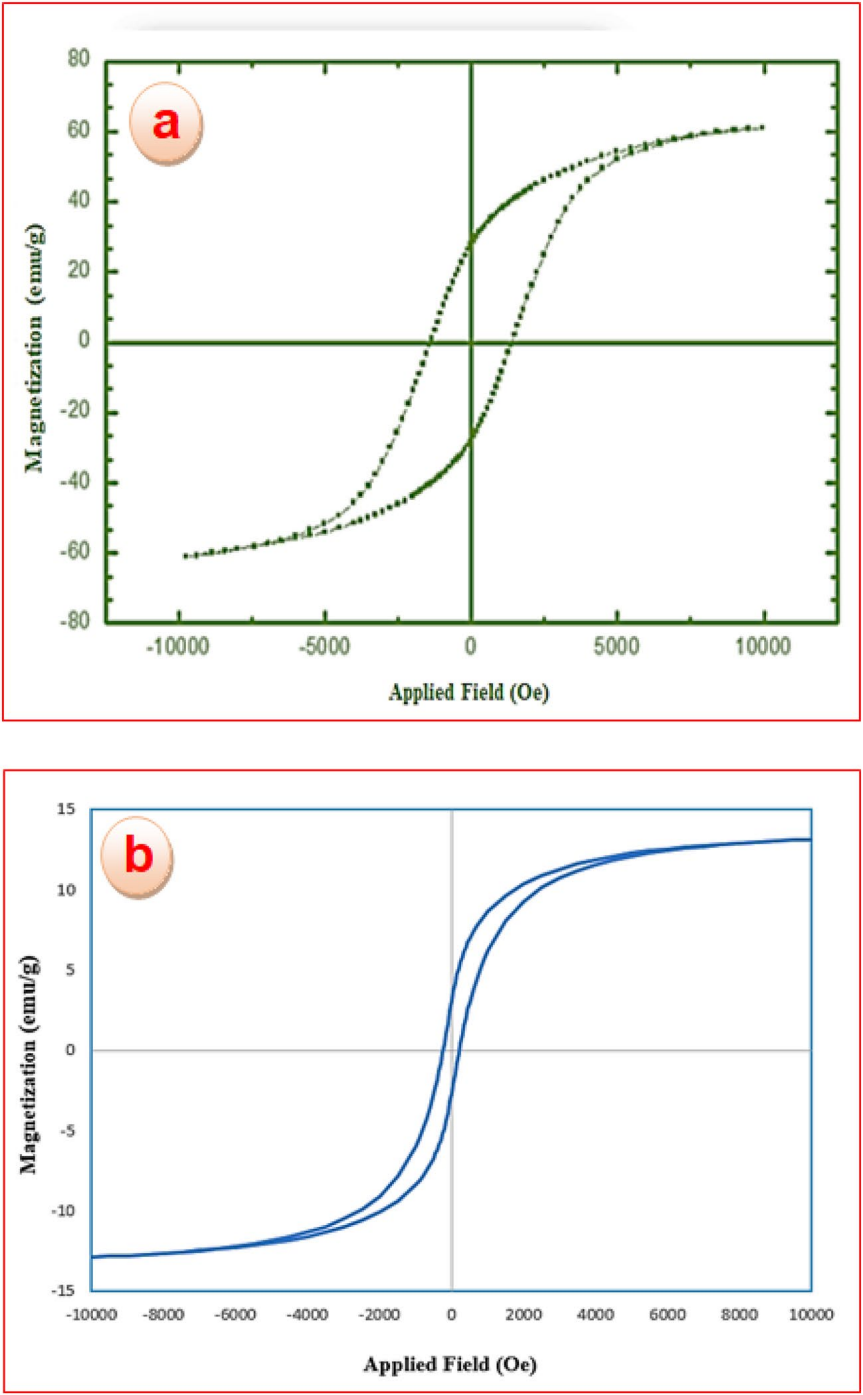
Magnetization curves of CoFe_2_O_4_ (**a**), CoFe_2_O_4_@calix-EDTA-Cs@PMA (**b**).

Magnetic parameters of CoFe_2_O_4_ nanoparticles (a) and CoFe_2_O_4_@calix-EDTA-Cs@PMA (b) were determined by vibrating sample magnetometer (VSM), the results are presented in a comparative manner in Fig. 3. Taking the results into account, saturated magnetization values for CoFe_2_O_4_ and CoFe_2_O_4_@calix-EDTA-Cs@PMA nanoparticles were recorded of 61.4 and 14.6 emu g^−1^, respectively. Compared with CoFe_2_O_4_ nanoparticles, a decrease of about 37.8 emu g^−1^ in saturation magnetization of CoFe_2_O_4_@calix-E0DTA-Cs@PMA can be attributed to the addition of the non-magnetic components.

#### EDX and elemental mapping analysis

**Figure 4:**
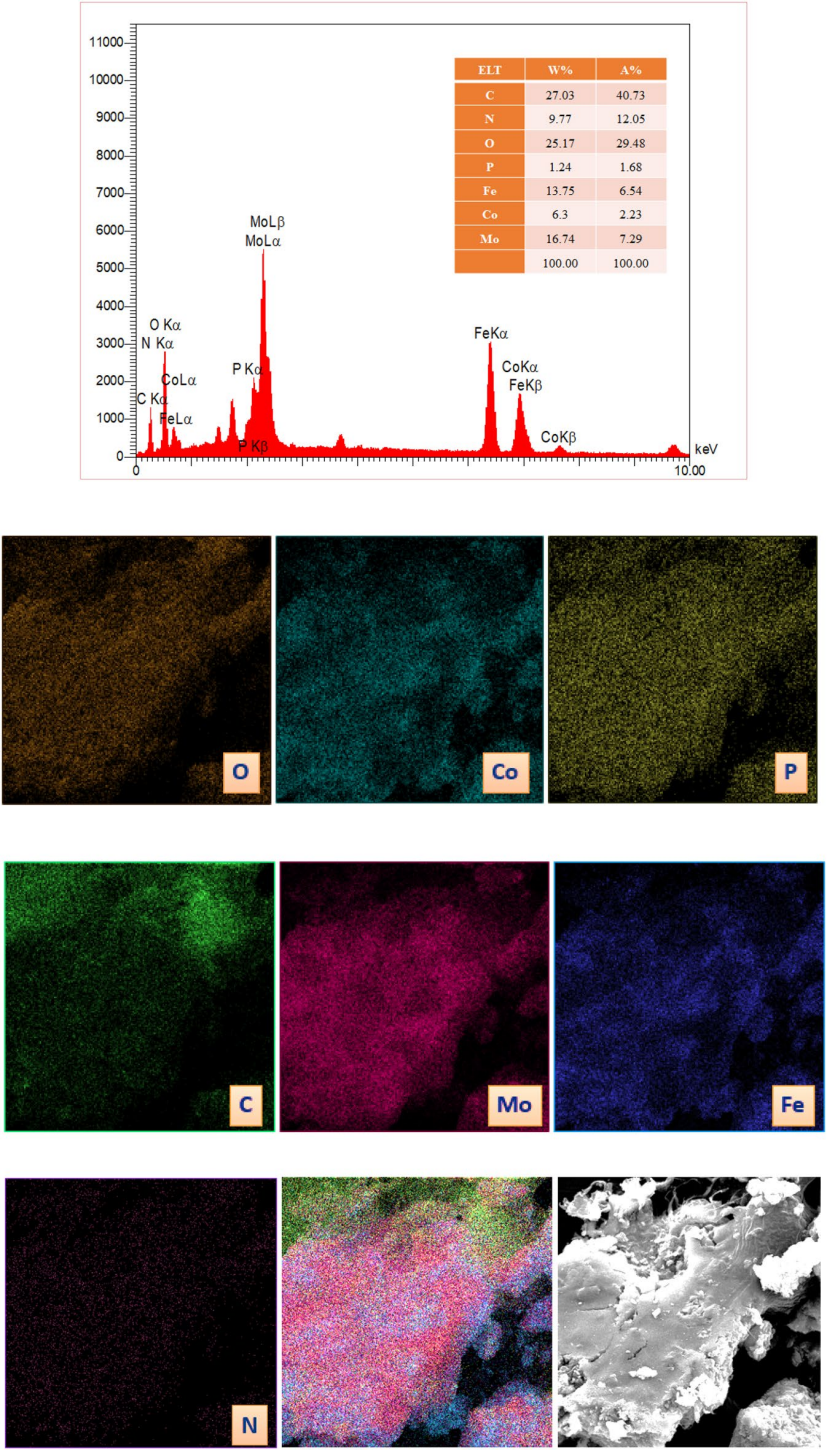
EDX spectrum and elemental mapping of CoFe_2_O_4_@calix-EDTA-Cs@PMA.

The confirmation of the presence of C, N, P, O, Mo, Co and Fe elements through EDX analysis indicates that our catalyst has been successfully synthesized. Moreover, the elemental mapping images results confirmed that the elements are uniformly distributed in the CoFe_2_O_4_@calix-EDTA-Cs@PMA network demonstrating. Even distribution in catalysts usually results in improving catalytic activity (Fig. 4).

The result of ICP-AES analysis of CoFe_2_O_4_@calix-EDTA-Cs@PMA showed that there are 0.34 mmol g^−1^ PMA in this synthesized catalyst.

The amount of H^+^ in the CoFe_2_O_4_@calix-EDTA-Cs@PMA determined by acid–base titration was 0.87 mmol g^−1^.

#### FE-SEM studies

**Figure 5:**
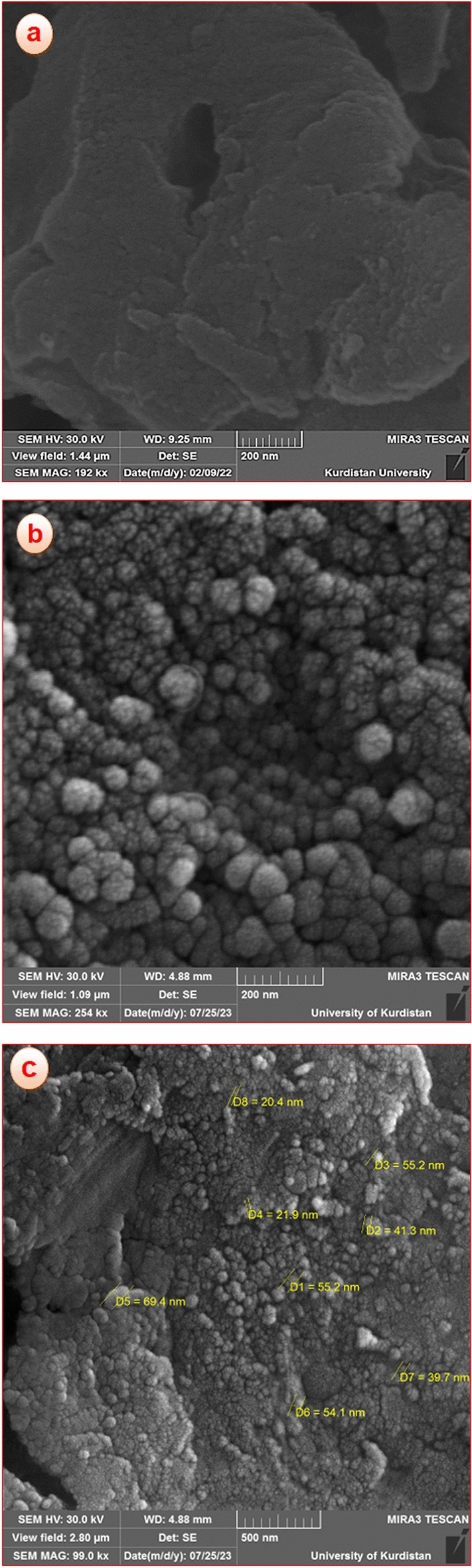
FE-SEM images of poly calix[4]resorcinarene at (**a**) 200 nm, CoFe_2_O_4_@calix-EDTA-Cs@PMA at (**b**) 200 nm, (**c**) 500 nm.

The FE-SEM is a useful technique utilized to analyze the morphology and particle size distribution of the synthesized nanoparticles. FE-SEM images of the poly calix[4]resorcinarene and the CoFe_2_O_4_@calix-EDTA-Cs@PMA catalyst are displayed in Fig. 5. As is evident, the poly calix[4]resorcinarene exhibit a spherical structure and uniformity (Fig. 5a). Furthermore, as shown in the image CoFe_2_O_4_@calix-EDTA-Cs@PMA comparing to the image of poly calix[4]resorcinarene, the SEM images confirm the successful anchoring of CoFe_2_O_4_ nanoparticles and the EDTA-Cs@PMA with dimensions underneath 100 nm on the surface of poly calix[4]resorcinarene (Fig. 5b,c).

#### Thermal gravimetric analysis

**Figure 6:**
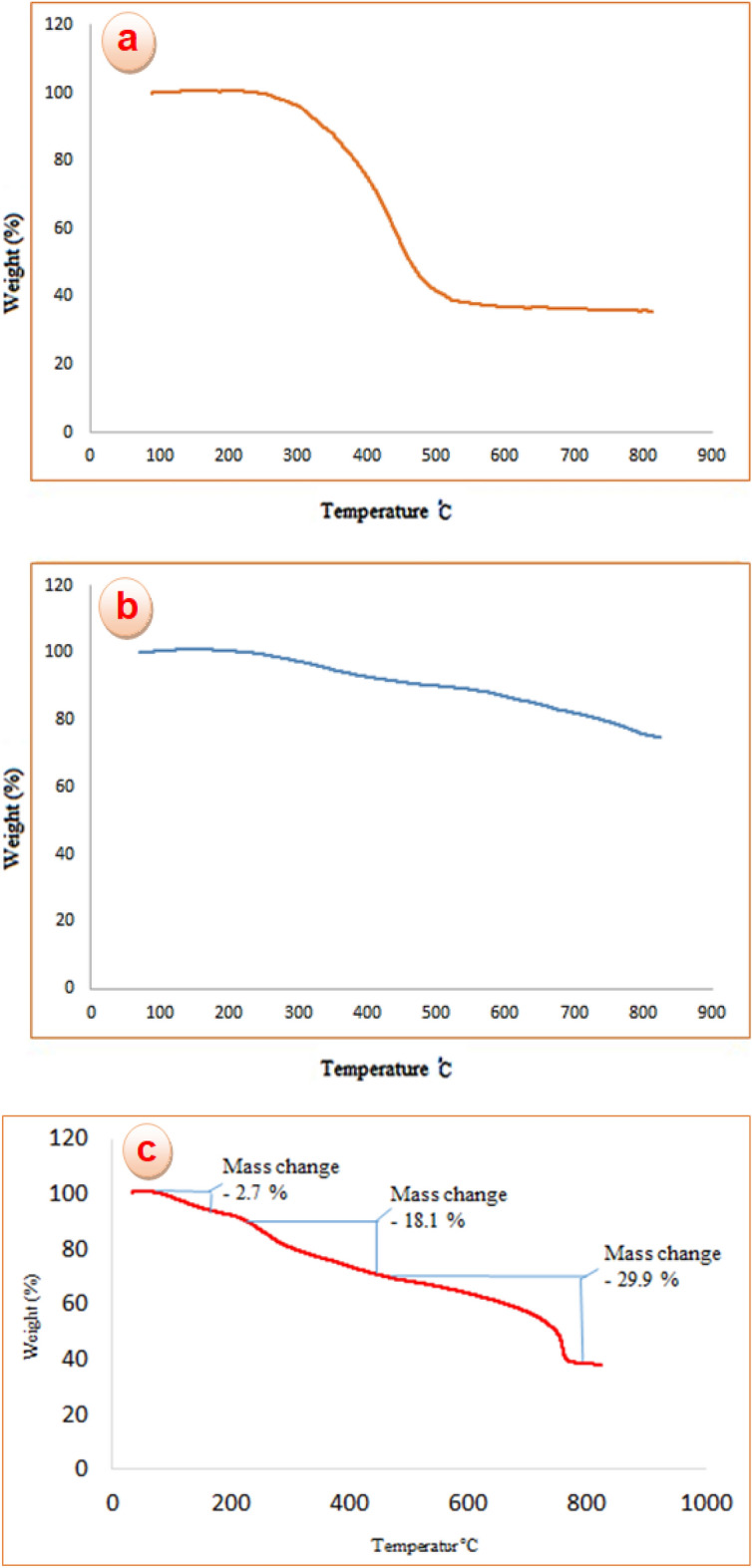
TGA curves of poly calix[4]resorcinarene (**a**), CoFe_2_O_4_@calix (**b**) and CoFe_2_O_4_@calix-EDTA-Cs@PMA (**c**).

To evaluate the thermal stability of the created catalytic system, TGA analysis for poly calix[4]resorcinarene (a), CoFe_2_O_4_@calix (b) and CoFe_2_O_4_@calix-EDTA-Cs@PMA (c) nanostructures were carried out in the temperature range of 25 to 850 °C (Fig. 6). The TGA curve displays that the thermal stability of poly calix[4]resorcinarene and CoFe_2_O_4_@calix are up to nearly 300 °C (Fig. 6a,b). A primary reduction in weight (2.7%) **at** 110–190 °C due to the elimination of physically adsorbed water takes place during the process of the catalyst mentioned. The second reduction in weight (18.1%) occurs within the temperature range of 220 to 460 °C and can be attributed to the decomposition of both chitosan and EDTA linker. Ultimately, an essential mass loss of about (29.9%) started from the region of 490 °C can be ascribed to the decomposition of the anchored polymeric units. This nanocatalyst demonstrates considerable thermal stability in a high-temperature setting (Fig. 6c).

#### AFM studies

**Figure 7:**
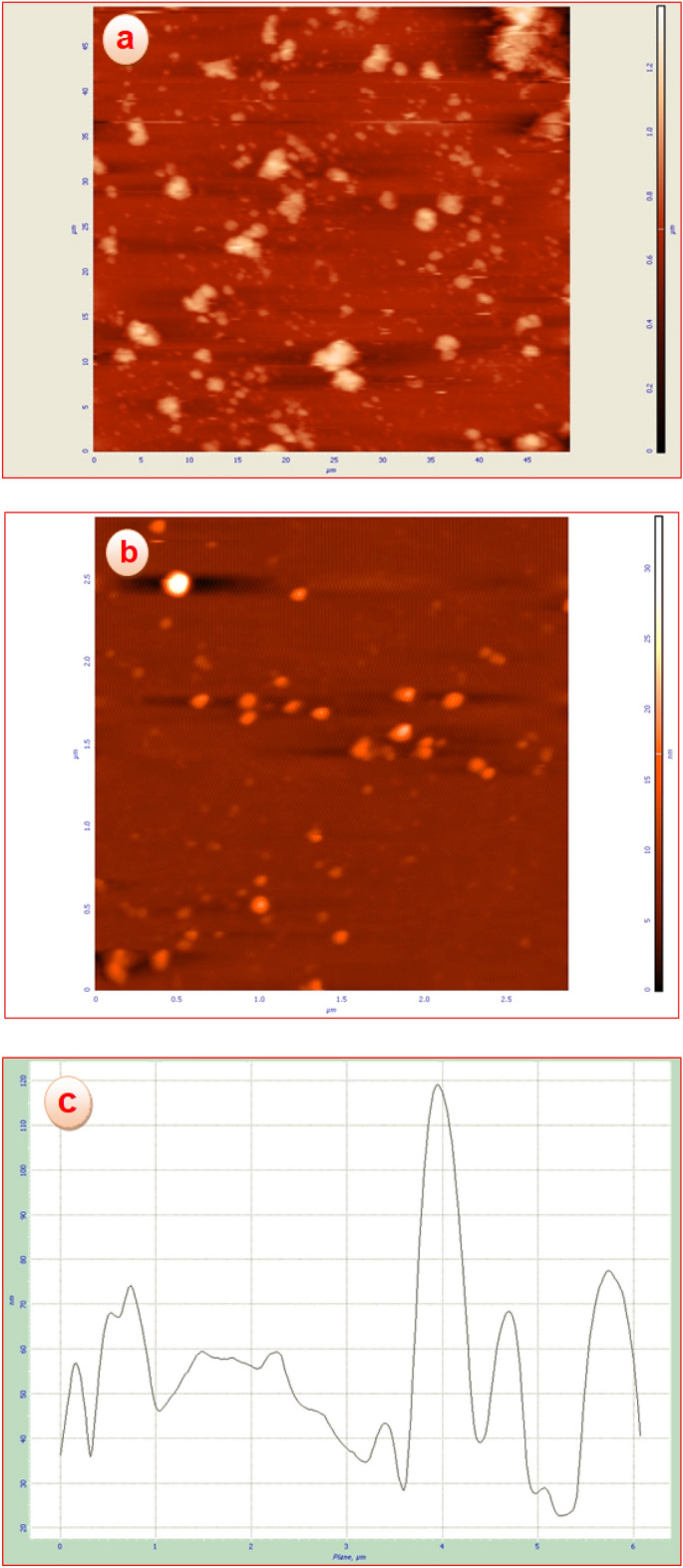
AFM images of 2D height and phase plots of (**a**) poly calix[4]resorcinarene, (**b**) CoFe_2_O_4_@calix-EDTA-Cs@PMA, and (**c**) an Image profile (the analysis of the height along a linear path) of CoFe_2_O_4_@calix-EDTA-Cs@PMA.

AFM atomic force microscope technique was used to obtain the morphology and the approximate size of surface cavities for poly calix[4]resorcinarene (a), and CoFe_2_O_4_@calix-EDTA-Cs@PMA (b, c) nanostructures (Fig. 7). According to the image and graph obtained for CoFe_2_O_4_@calix-EDTA-Cs@PMA, structures containing pores and porosity can be well recognized at the nano scale. The existence of smaller pores (about 60 nm) in the CoFe_2_O_4_@calix-EDTA-Cs@PMA compared to the primary polymer (about 311 nm) can be proof of the successful process of functionalization and synthesis of CoFe_2_O_4_@calix-EDTA-Cs@PMA catalyst.

#### N_2_ adsorption–desorption isotherms studies

**Figure 8:**
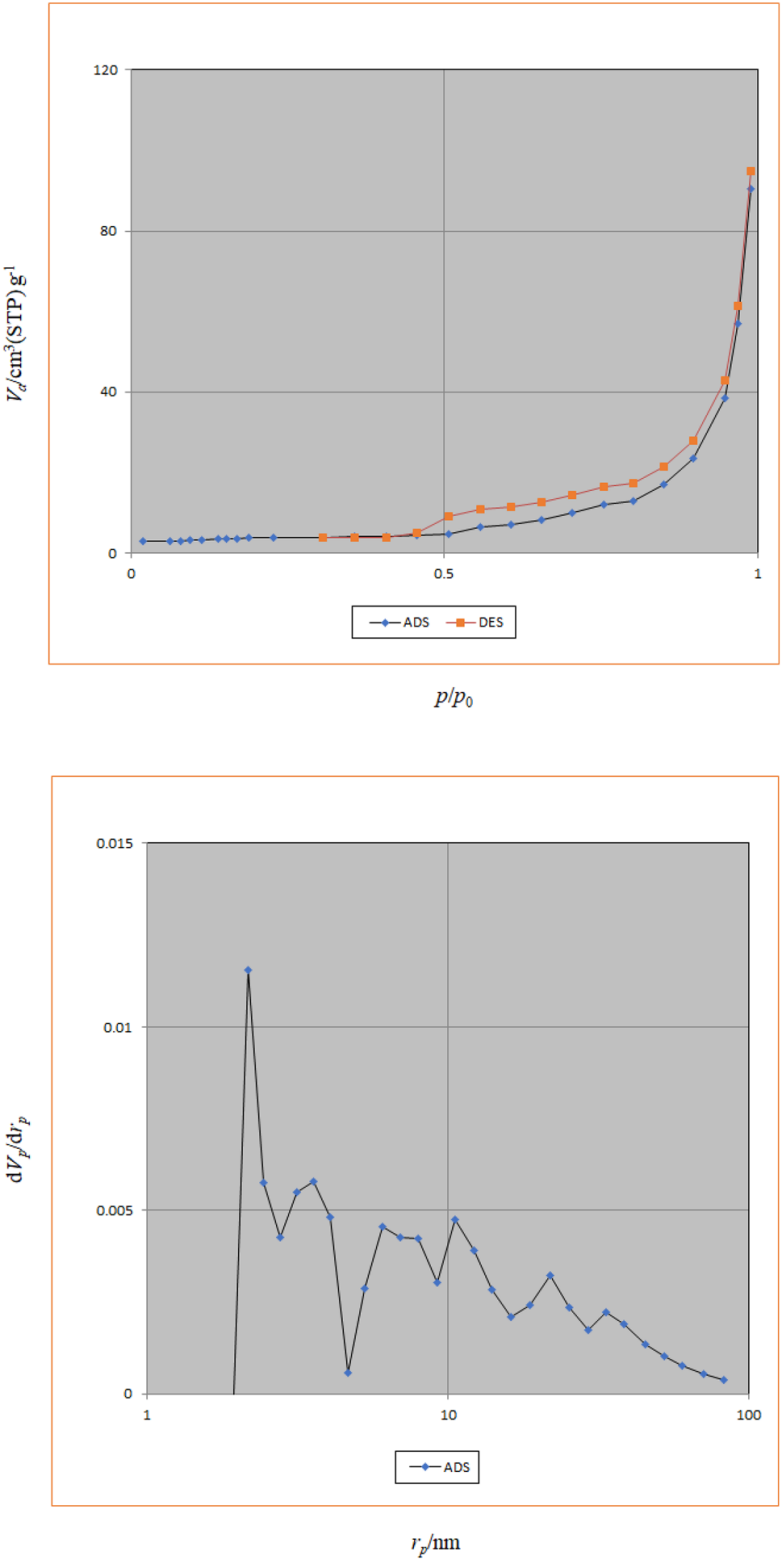
Nitrogen adsorption–desorption isotherms and BJH pore size distributions of CoFe_2_O_4_@calix-EDTA-Cs@PMA.

The nitrogen adsorption–desorption isotherms and pore size distributions of CoFe_2_O_4_@calix-EDTA-Cs@PMA is illustrated (Fig. 8). The materials had type IV isotherms, indicating that the mesostructure remained. According to Brunauer–Emmett–Teller (BET) analysis, the surface area, the pore volume, and the pore size of the catalyst are 13.27 m^2^ g^−1^, 0.14 cm^3^ g^−1^, and 42.66 nm, respectively. The results show that the magnetic particles are stuck together and reduce the contact surface for the adsorbed gas and adsorbent material to collide, which reduces the specific surface area of this compound and its less tendency to absorb the adsorbed material. In addition, due to the placement of chitosan layers on nanoparticles, it leads to the polymer state of the structure.

### Catalytic studies

For evaluation of its applicability in organic reactions, these acidic magnetic nanoparticles were utilized in the preparation of NH-1,3-oxazolidine-2-ones derivatives, initially, to optimize the reaction of α-epoxyketone with NaOCN in the presence of CoFe_2_O_4_@calix-EDTA-Cs@PMA as model substrate was tested. Various reaction conditions, including the solvent, catalyst quantity, and temperature, were analyzed for their impact.

To determine the optimal reaction temperature, this process was examined at different temperatures and 50 °C was found to be the most effective under ultrasonic conditions (Table 1). The reaction yield was trace at room temperature (Table 1, entry 10). Additionally, the output of the reaction was fixed at temperatures more than 50 °C (Table 1, entry 8).

The reaction was repeated in different solvents (Table 1, entries 1–6), it was found that PEG-400 is more suitable for this reaction and the nonpolar solvents provided the lowest conversion. The reaction proceeded with high speed and the corresponding products were isolated in excellent yields with 25 mg of the catalyst. In the absence of acid catalyst, no reaction was observed (Table 1, entry 14), indicating that the CoFe_2_O_4_@calix-EDTA-Cs@PMA is necessary for the reaction. Additionally, the reaction was carried out with CoFe_2_O_4_ as a catalyst, but it did not result in any progress even after an extended period (Table 1, entry 16).

With the optimized conditions, the reactions of different α-epoxyketones were tested (Table 2).

**Table 2:** The preparation of NH-1,3-oxazolidin-2-one derivatives in the presence CoFe_2_O_4_@calix-EDTA-Cs@PMA.


Substrate		Time (min)	Yield (%)	Conversion (%)^a^	Ratio^b^	
					*cis*	*trans*
a		30	95	99	65	35
b		25	93	99	59	41
c		20	91	99	70	30
d		20	93	95	90	Trace
e		10	88	97	30	70
f		10	91	97	45	55
g		40	85	90	55	45
h		45	83	85	53	47
I		60	50	72	51	–
J		45	88	90	74	26
K		90	–	–	–	–
l		90	–	–	–	–
m		90	–	–	–	–

^a^Based on consumed α-epoxyketones.

^b^Isolated yield.

It is evident from the data in Table 2, that cis isomers are found to be the major product also the rates and output of reaction were enhanced in the presence of electron-donating groups instead of Y.

**Figure 9:**
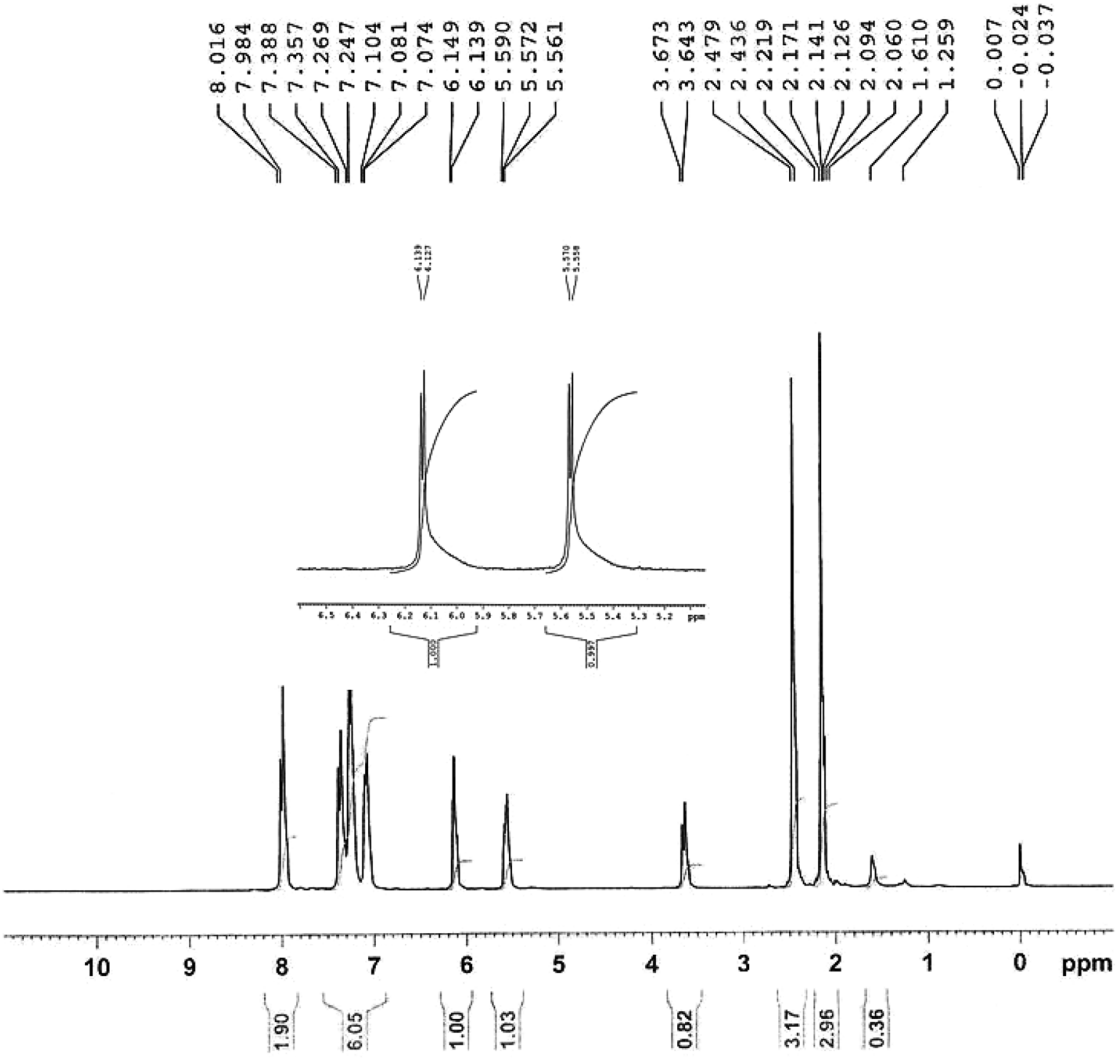
^1^H NMR spectrum of cis-5-(4-methylbenzoyl)-4-(4-methylphenyl) oxozolidin-2-one.

The ^1^H NMR spectrum in CDCl_3_ of cis-5-(4-methylbenzoyl)-4-(4-methylphenyl) oxozolidin-2-one (cis-2c, C_16_H_13_NO_3_) are displayed in Fig. 9. In the ^1^H NMR spectrum, the NH-signal of the proton of the oxazolidin-2-one ring is δ = 3.5–4.2 instead of δ = 6–7 ppm. Probably, the aryl group is perpendicular to the NH-bond, so the shielding effect of the aryl group causes the NH-band to appears at higher field. Also, the signals of the protons CH–C–O, CH–C–N are δ = 6.25, 5.58 ppm respectively and the singles of the of aryl and aryl groups appears at about 7–8.

**Scheme 2:**
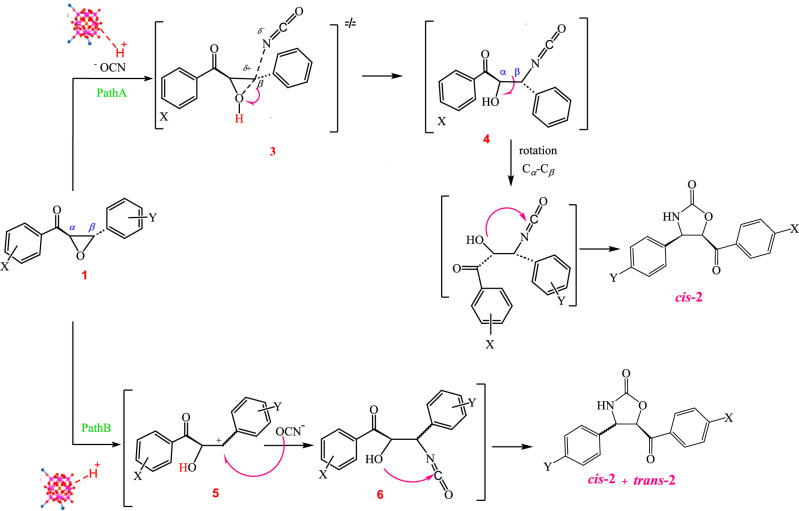
Proposed mechanism for the synthesis of NH-1,3-oxazolidin-2-one, of α-epoxyketone with NaOCN in presence CoFe_2_O_4_@calix-EDTA-Cs@PMA.

Drawing from past studies^37^ and our own findings during this reaction, we can suggest the following mechanism, which involves two pathways (Scheme 2). According to this mechanism; in path **A**, the epoxide ring of α-epoxyketone is first activated by coordination to PMA on the surface of CoFe_2_O_4_@calix-EDTA-Cs@PMA nanocatalyst subsequently sodium cyanate attack the epoxide ring to produce transition state **3**. After the opening of the epoxide ring, intermediate **4** is formed. Fast rotating the C_*a*_–C_*b*_ bond and cyclization of intermediate **4** under the reaction conditions leads to the NH*-*oxazolidine-2-ones **2**. In path **B**, a carbocation **5** is initially formed by the acidic catalyst. Subsequently, a nucleophilic attack by sodium cyanate on this intermediate leads to the formation of **6** and cyclization of intermediate state **6** may result in trans and cis isomers. Since cis isomers are the main products of the reaction, the most liable mechanism is the generation of transition state **3** through pathway **A**. Since cis isomers are the main products of the reaction, the most liable mechanism is the generation of transition state **3** through pathway **A**. The reaction proceeded with high regio-, chemo- and stereoselective, and the carbonyl group of the ketone in α-epoxyketone remained without any change.

### Comparison of the catalyst

A brief comparison between the efficiency of the present catalyst and some of those previously reported ones in the literature is listed in Table 3. The results show that the current research can be described as a highly effective catalyst due to its easy separation, high yield, fast reaction time, and low reaction temperature.

**Table 3:** Comparison of catalytic activity of CoFe_2_O_4_@calix-EDTA-Cs@PMA nanocatalyst with some recently reported procedures.

Entry	Catalyst	Reaction condition	Solvent	Time (h)	Yield (%)	Ref.
1	CoFe_2_O_4_@calix-EDTA-Cs@PMA	Sonication/50 °C	PEG-400	30 min	95	This work
2	HNTf_2_	r.t	DCM	55	62	^42^
3	NaH	25–30 °C	DCM//DMF	9	92	^43^
4	ClCO_2_CH_3_	Reflux	CH_3_CN	12	87	^44^
5	MCPBA	65 °C	THF/DMF	4	84	^45^

### Recycling of CoFe_2_O_4_@calix-EDTA-Cs@PMA

**Figure 10:**
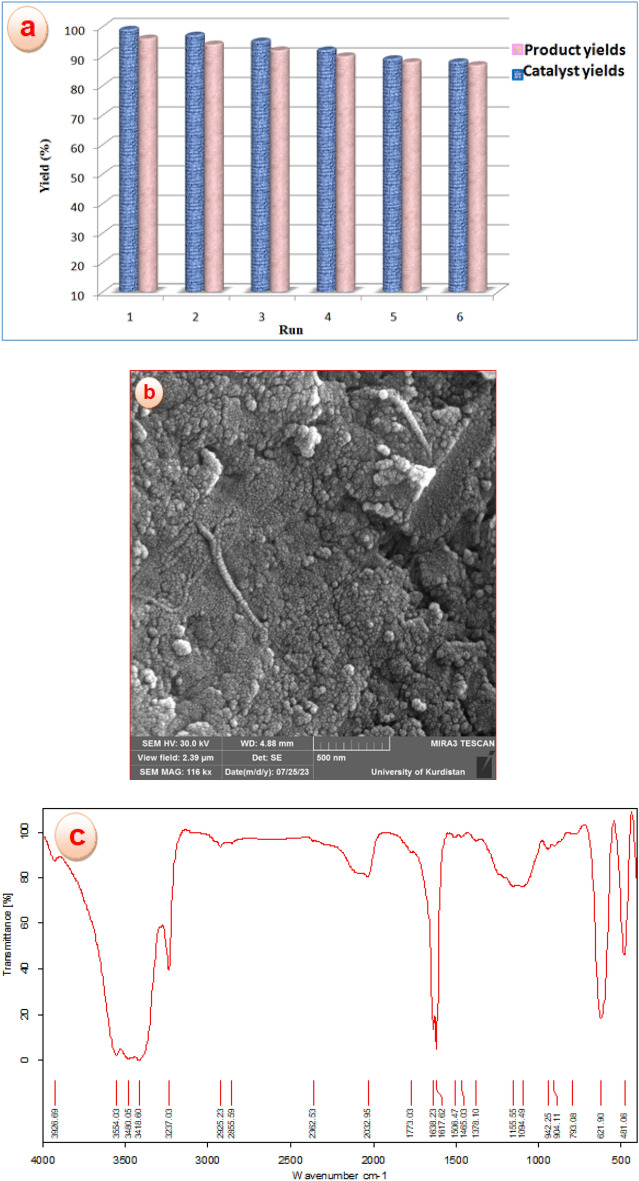
Reusability of CoFe_2_O_4_@calix-EDTA-Cs@PMA for the synthesis of NH-oxazolidin-2-one, (**a**) analyses of reused catalyst six runs, (**b**) FE-SEM and (**c**) FT-IR.

For studying the recyclability of CoFe_2_O_4_@calix-EDTA-Cs@PMA, the catalyst was filtrated using a magnetic field, then rinsed with CH_2_Cl_2_, and dried and applied for the next run of the reaction. The catalyst was recovered for a subsequent 6 cycles without considerable loss of its catalytic activity (Fig. 10a). FT-IR and FE-SEM analyses of reused catalyst after sixth run illustrated that no significant changes of the nanocomposite occurred during the reaction (Fig. 10b,c).

In addition, to determine the degree of leaching of the metal from the heterogeneous catalyst, the catalyst was removed by using a magnetic field and the molybdenum amount in reaction medium after each reaction cycle was measured through Inductively Coupled Plasma (ICP) analyzer. The analysis of the reaction mixture by the ICP technique showed that the leaching of H_3_PMo_12_O_40_ was negligible.

The recovery and reusability of catalyst were in-vestigated for the preparation of *cis*-5-Benzoyl-4-(4-methylphenyl)oxazolidin-2-one (2b) under optimized conditions (Table 4).

**Table 4:** Recyclability CoFe_2_O_4_@calix-EDTA-Cs@PMA in the synthesis of NH-1,3 oxazolidin-2-one under the optimized conditions and PMA leaching (%) in reaction.

Run	1	2	3	4
Yield (%)^a^	99	97	93	90
Leaching (%)	0.40	0.72	1.09	1.43

^a^Reaction conditions: α-epoxyketone (1 mmol), sodium cyanate (1 mmol), CoFe_2_O_4_@calix-EDTA-Cs@PMA (25 mg), solvent (4 mL), for 25 min.

## Conclusions

The current study has presented the novel magnetic nanoparticles of CoFe_2_O_4_@calix-EDTA-Cs@PMA. This catalyst is both acidic and magnetic, which enables its facile separation via an external magnet. For evaluation of its applicability in organic reactions, this acidic magnetic nanoparticle was utilized in the preparation of 5-Aroyl-NH-1,3-oxazolidin-2-ones derivatives via reaction of α-epoxyketones with NaOCN under the ultrasonic irradiation conditions in PEG-400 solvent. The green protocol is attractive in terms of its simplicity of procedure, high yields, environmental compatibility, easy separation of the catalyst, recycle exploitation and good to excellent isolated yields. So, we think that the design protocol could be considered a new and useful addition to the present methodologies in these scopes.

## Data Availability

The datasets used and/or analysed during the current study available from the corresponding author on reasonable request.
